# Positive recovery for low-risk injuries screened by the short form - Örebro musculoskeletal pain screening questionnaire following road traffic injury: evidence from an inception cohort study in New South Wales, Australia

**DOI:** 10.1186/s12891-019-2881-9

**Published:** 2019-11-13

**Authors:** Ha Nguyen, Trudy Rebbeck, Annette Kifley, Jagnoor Jagnoor, Michael Dinh, Amith Shetty, Michael Nicholas, Ian D. Cameron

**Affiliations:** 1 John Walsh Centre for Rehabilitation Research, The University of Sydney, Sydney, Australia; 2 Injury Division, The George Institute for Global Health, Sydney, Australia; 3 Australian Centre for Precision Health, The University of South Australia, Adelaide, Australia; 4 NSW Institute of Trauma and Injury Management, Sydney, Australia; 5 Emergency department, Westmead Hospital, Sydney, Australia; 6 Pain Management Research Institute, Royal North Shore Hospital, The University of Sydney, Sydney, Australia

**Keywords:** Road traffic injury, Prognosis, Risk stratification, Recovery, Global perceived effect, Return to work

## Abstract

**Background:**

Prognosis of musculoskeletal disorders following injury is essential in determining appropriate treatment and care. A generic validated prognostic tool to stratify risk of poor recovery for people with musculoskeletal injuries after road traffic crash is not available. This study aimed to examine differences in recovery, return to work and health related quality of life between low and high-risk of poor recovery people with musculoskeletal injuries stratified by the Short form - Örebro Musculoskeletal Pain Screening Questionnaire (SF-OMPSQ).

**Methods:**

In an inception cohort study, participants with non-fracture musculoskeletal injury with the main site being the neck, lower back or lower limb were stratified into low (score ≤ 50) and high (score > 50) risk of poor recovery using the SF-OMPSQ score at baseline. We assessed the proportion of fully recovered participants (Global Perceived Effect scale ≥4), the proportion returning to work and changes in short form 12-item (SF-12) scores between baseline and 6-month follow-up in low and high-risk groups. Modified Poisson regression was used to estimate the adjusted risk ratio (RR) of being recovered and return to work in the low and high-risk groups. Paired t-test was used to compare changes in SF-12 physical and mental component summary scales, and chi-square test was used to assess the significance of the risk ratio of fully recovered between low and high-risk groups.

**Results:**

The study included 498 participants (166 with neck, 78 with lower back and 254 with lower limb injuries). The proportion of being recovered was significantly higher in the low than the high-risk groups (Adjusted risk ratio: 2.96 [95% CI: 1.81 to 4.82]). Significantly more people in the low-risk group returned to work (91.0%) than the high-risk group (54.6%). People at low-risk had higher SF-12 scores at baseline and 6-month follow-up than those at high-risk. There were no differences between injury types for recovery and return to work at 6 months.

**Conclusion:**

The SF-OMPSQ could be recommended as a generic prognostic tool to identify individuals with musculoskeletal injuries early after road traffic injury, who would have a higher or lower likelihood of recovering or returning fully to pre-injury work.

**Trial registration:**

Australia New Zealand Clinical trial registry identification number - ACTRN12613000889752. Registered 09 August 2013.

## Background

Road traffic injury (RTI) is a major public health problem worldwide, contributing to a large burden of mortality, disability and economic loss. According to the World Health Organisation Global status report on road safety, RTI claims over 1.2 million lives and costs governments nearly 3% of the GDP [1]. In Australia fatal RTIs is decreasing [2], however non-fatal RTIs and their associated costs remain significant. The 2015 Australian Institute of Health and Welfare on serious injuries due to road traffic crashes shows from 2001 to 2010, there was an average annual increase of 0.9% (from 141.6 to 146.6 per 100,000 population) [3]. In 2010, the Bureau of Infrastructure, Transport and Regional Economics estimated the social cost of road crashes to Australia was $17.85 billion in 2006, equivalent to approximately 1.7% of the total GDP [4]. Large proportion of the costs was associated with factors such as injury treatment and rehabilitation, disability and loss of productivity.

Prognosis of musculoskeletal disorders following injury is essential in determining appropriate treatment and care. People with poor prognosis often undergo unnecessary care and treatment, which contribute to significant personal, economic and social burden associated with the condition [5, 6]. In the area of musculoskeletal health care, there is increasing interest in developing and applying prognostic screening tools to stratify patients into risk levels of recovery to direct appropriate level of care. Essentially, those with higher risk should receive more comprehensive care than those with lower risk. To date there is evidence that stratified care has improved outcomes using condition specific tools. For instance, use of the Keele STarT Back Screening Tool to provide stratified treatment for patients with low back pain has demonstrated clinical and cost effectiveness in the UK [7]. Similarly, the short form Orebro Musculoskeletal Pain Screening Questionnaire (SF-OMPSQ) [8] was used to direct appropriate care for workers with soft tissue injury, demonstrating clinically significant improvements in outcomes such as disability and sustained return to work [9]. The SF-OMPSQ covers concept areas found to be associated with recovery, including self-reported level of pain, self-perceived function/disability, distress, fear avoidance and recovery expectation [8]. These concept areas are also among the priority measures for inclusion recommended for future prognostic studies for whiplash injury [6]. Recently, Rebbeck and colleagues conducted a randomised trial [10] to evaluate stratified care for people with whiplash associated disorder using a validated clinical prediction rule [11]. In addition to these studies, there are many prognostic tools to identify the risk of non-recovery early after presentation for specific musculoskeletal injuries such as whiplash [12–15], idiopathic neck pain [16, 17], low back pain [18], musculoskeletal pain and knee osteoarthritis patients [19, 20]. To date however, there have been no studies that have evaluated a tool that accurately stratifies risk across common musculoskeletal injuries following a road traffic crash.

For clinicians, it is more acceptable and more likely to be used if a single validated prognostic tool could stratify risk of non-recovery across common musculoskeletal injuries. Given the positive outcome from the use of the SF-OMPSQ to drive care for injured workers and its recommendation in recent published models of care [21], we investigated the application of the SF-OMPSQ to stratify risk of non-recovery for people with musculoskeletal injuries after RTI. Specifically, we aimed to examine differences in recovery, return to work and health related quality of life between low and high-risk of poor recovery people with common musculoskeletal injuries (neck, low back and lower limb) at 6 months after RTI.

## Method

### Study design and participants

This is an inception cohort study with participants sustained acute musculoskeletal injuries from the Study on factors influencing social and health outcomes following road traffic injuries in New South Wales (NSW), Australia (the FISH study). Participants were eligible for the FISH study if they were at least 17 years old, English speaking, NSW resident, injured in a motor vehicle crash on land diagnosed within 28 days by a medical/registered health practitioner. Ineligible participants were those who were injured involving non-motorised vehicle, with severe injury (e.g. severe traumatic brain injury, spinal cord injury, excessive burn, or multiple amputations), with isolated, superficial soft tissue injuries (e.g. bruises, abrasions, or cuts), intentional self-harm or fatal injuries. In the FISH study, participants were recruited between July 2013 and December 2016 from the emergency departments of eight metropolitan hospitals (Canterbury, Concord, John Hunter, Liverpool, Royal Prince Alfred, Royal North Shore, St George and Westmead hospitals), three rural NSW health services (Orange, Dubbo and Bathurst), primary care and the NSW State Insurance Regulatory Authority – Personal Injury Registry, and Claims Advisory Service. Further details on sample size, participant recruitment and ethics approvals are described elsewhere [22]. For the present study, we only included the FISH study participants with non-fracture musculoskeletal injury with the main site being the neck (whiplash), lower back or lower limb.

### Data collection and data items

Data were collected at baseline (within 28 days of the crash) and at 6-month follow-up. At baseline, a trained research assistant gained informed consent via telephone, and then conducted the baseline assessment following a structured process using Computer Aided Telephone Interview. Outcomes were assessed 6 months after the injury by telephone, mail or email.

Data collected at baseline include participants’ demographic (e.g. age and gender), socio-economic characteristics (e.g. employment status and income group), and circumstances of the injury and crash. The questionnaires administered at baseline also include items on general health status pre and post-injury, health related quality of life (the 12-Item Short Form Health Survey, SF-12 [23]) and the short form OMPSQ (SF-OMPSQ) [8]. At 6 months follow-up, participants were contacted to update their socio-economic, global perceived recovery, returning to work status and health related quality of life (SF-12).

### Risk stratification at baseline

The short form 10-item OMSPQ was used as a tool to stratify risk of non-recovery into low and high level at baseline. It was derived from the original 25-item OMSPQ, a validated tool that assists clinicians in identifying people with musculoskeletal injury at risk of persistent pain [24–26]. The short version was developed with greater clinical utility by being shorter and easier to administer and score than the original; it was found to be nearly as accurate as the long version [8]. Participants with a score of greater than 50 (out of a total of 100) were identified as “high-risk” of poor recovery and those with a score of 50 or less were “low-risk” similar to Gopinath et al.’s studies [27, 28].

We used an adapted version of the published SF-OMPSQ. Our version included 10 questions, including six from the published SF-OMPSQ, and four on pain, self-perceive function, sleep and distress which mirror the same concept and structure of the published SF-OMPSQ. We included eight of the ten questions. The two additional questions were assessed from responses available in other questionnaires, including the sleep question (“I can sleep at night”) replaced by the one in the Impact of Event Scale (“I had trouble falling asleep”), and the tension/anxiety question (“How tense or anxious have you felt in the past week”) replaced by the stress subscale score of the Depression Anxiety Stress Scale. These were rescaled to that they ranged from 0 to 10 to be the same as the equivalent items in the SF-OMPSQ. This adapted version was also used Gopinath et al.’s study to identify prognostic indicators of social outcomes [27] after RTIs and health related quality of life [28]. The tool was found to be able to discriminate people with low-risk of poor recovery (score ≤ 50) from those with high-risk (score > 50). Compared to high-risk of poor recovery people, those with low-risk had significantly higher likelihood to return to work, resume to full duties at work [27], and higher quality of life scores [28].

### Measurements of outcome

The primary outcome was recovery measured by the Global Perceived Effect (GPE) at 6 months. The GPE asks patient to rate how much their condition has improved since the injury on a scale ranging between − 5/5 (vastly worse), 0 (unchanged) and + 5/5 (completely recovered). The GPE was found to be reliably rated by patients with musculoskeletal conditions [29]. In this study, we considered recovery as GPE ≥4 on the scale and non-recovery as GPE < 4, similar to other studies of participants with whiplash injuries [30, 31].

The secondary outcome measures were return to work and health related quality of life at 6 months. In 6-month follow-up interview, participants were also asked the impact of the injury on their work. Work status was evaluated as whether they returned to paid-work at the same level prior to the injury by asking participants “Whether they had returned to work since the accident?”. If they were working, “what was their employment status?” with response options being paid work, self-employed or non-paid work; and whether it was “full duties” or “modified duties, e.g. lifting restrictions, reduced hours”. Health related quality of life was measured by the 12-item Short Form Health Survey (SF-12), which has been widely used in many research and population surveys, including injury specific studies [28, 32, 33]. SF-12 was summarised into two component scores, the Physical Component Summary (PCS) and the Mental Component Summary (MCS) scales. The two scales range between 0 and 100, with higher value indicating better health.

### Statistical analyses

Modified Poisson regression was used to estimate the adjusted risk ratio (RR) of being recovered (GPE at 6 months ≥4) and the adjusted RR of being returning fully to pre-injury work between the low and high-risk groups. Modified Poisson regression is a regression applied to binomial data using a robust error variance and is found to estimate RR consistently and efficiently [34]. Participants’ characteristics to be adjusted were those found to be statistically significantly associated with the outcomes of interest (i.e. being recovered or returning to work at 6-month after the injury) in univariable analyses. Characteristics that were assessed for association with outcomes sex, categories of education level (secondary and post-secondary), occupation (white and blue collar), paid work status (yes and no), annual income (loss or more than AU$ 65,000 per annum), smoking status (yes and no), alcohol use (weekly or more and monthly/never), BMI (obese/overweight and normal), pre-injury chronic illness (yes and no), road user group at the time of accident. We also included characteristics which were statistically significantly associated with recovery in prior studies [15, 27, 28, 35], such as age, self-rated general health and hospital admission status following injury.

Paired t-tests were employed to examine the change in health-related quality of life score (SF-12 physical and mental component summary scales) at baseline and 6-month follow-up within the same injury group.

A *p*-value of less than 0.05 was considered statistically significant. All analyses were performed using STATA statistical package version 12 (Stata Corporation, College Station, TX, USA).

## Results

### Characteristics of participants

The present study includes 498 people (166 with neck, 78 with lower back and 254 with lower limb injuries). Across injury groups at baseline, characteristics with no statistically significant differences across three groups of injury include age, education, occupation category, paid work, income groups, alcohol use groups and BMI groups (Table 1). The proportion of males in the lower limb injury group (69%) was significantly higher than that in the neck injury group (36%) (*p* <  0.001). There were significantly more people with low back pain who smoked (26%) compared with those with neck pain (11%) and lower limb injures (17%; *p* = 0.019). Finally a higher proportion of those with lower limb injury had pre-injury chronic illness (83%) compared with lower back injury group (26%; *p* = 0.014).

**Table 1:** Socio-demographic and lifestyle characteristics of study participants at baseline and 6-month follow-up

	Baseline			Lost to follow-up at 6 months		
	Neck (*n* = 166)	Lower back (*n* = 78)	Lower limb (*n* = 254)	No (*n* = 347)	Yes (*n* = 151)	*p*-value
Age (mean, SD)	40.2 (16)	35.7 (17)	38.9 (15)	39.4 (16)	37.6 (16)	0.26
Males	59 (36%)	47 (60%)	176 (69%)	204 (59%)	78 (52%)	0.14
Post-secondary education	113 (68%)	45 (58%)	155 (61%)	231 (67%)	82 (54%)	0.01
Occupation						
White collar	107 (64%)	37 (47%)	133 (52%)	193 (56%)	84 (56%)	0.56
Blue collar	21 (13%)	19 (24%)	60 (24%)	66 (19%)	34 (23%)	
Missing	38 (23%)	22 (28%)	61 (24%)	88 (25%)	33 (22%)	
Paid work	128 (77%)	56 (72%)	194 (76%)	260 (75%)	118 (78%)	0.44
Annual income						
$65,000 or less	69 (42%)	26 (33%)	89 (35%)	118 (34%)	66 (44%)	0.08
$65,000 or more	54 (33%)	26 (33%)	100 (39%)	135 (39%)	45 (30%)	
Missing	43 (26%)	26 (33%)	65 (26%)	94 (27%)	40 (26%)	
Smoking						
Current smoker	19 (11%)	20 (26%)	42 (17%)	46 (13%)	35 (23%)	0.01
Alcohol use						
Weekly or more	74 (45%)	36 (46%)	134 (53%)	177 (51%)	67 (44%)	0.57
BMI group						
Overweight/Obese	80 (48%)	46 (59%)	139 (55%)	185 (53%)	80 (53%)	0.48
Pre-injury chronic illness						
Yes	112 (67%)	43 (26%)	137 (83%)	208 (60%)	84 (56%)	0.35

Note: *SD* Standard deviation, *BMI* Body mass index

**Fig. 1:**
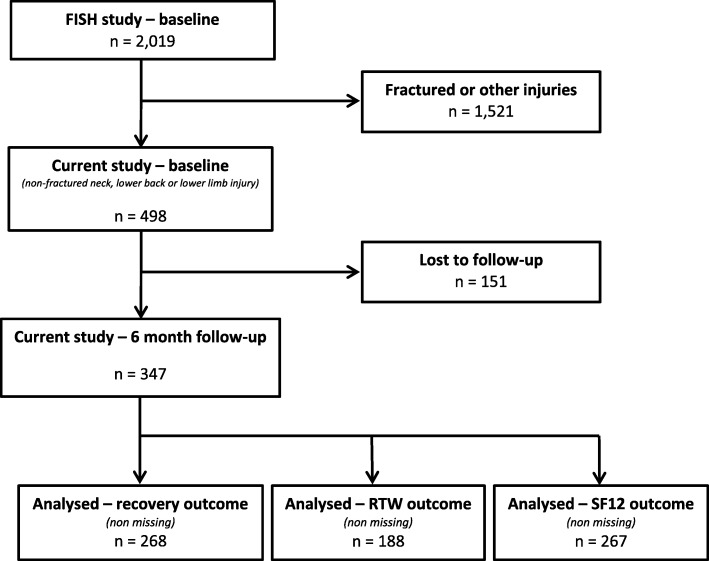
Flowchart of study participants

At 6-month follow-up, 70% (347/498) participants remained in the study (Fig. 1). Comparisons across socio-demographic and lifestyle characteristics for participants who were followed-up and not followed-up at 6-month indicate that majority of them were not statistically significant. Characteristics with statistically significant differences were the proportion of post-secondary education (lower in the lost to follow-up group) and the proportion of smoker (higher in the lost to follow-up group).

### Injury and risk stratification

Injury characteristics at baseline and 6-month follow-up are presented in Table 2. A significantly greater proportion of those who sustained a neck injury were drivers (73%), compared with those sustained a lower limb injury (20%). Of those with lower limb injury, most sustained this as a result of a motorcycle accident (50%) and this group had significantly more hospital admissions (52%) compared with 44% among lower back and 31% in the neck injury groups. In terms of risk stratification at baseline, significantly more people with lower limb injures were stratified as low-risk of non-recovery (57%) compared to 41% of those with lower back injuries.

**Table 2:** Some injury characteristics of study participants

	Baseline			Lost to follow-up at 6 months		
	Neck (n = 166)	Lower back (n = 78)	Lower limb (n = 254)	No (n = 347)	Yes (n = 151)	*p*-value
Role at the time of the accident						
Driver	121 (73%)	43 (55%)	50 (20%)	134 (39%)	80 (53%)	< 0.01
Passenger	30 (18%)	11 (14%)	10 (4%)	32 (9%)	19 (13%)	
Motorcycle rider	8 (5%)	15 (19%)	128 (50%)	118 (34%)	33 (22%)	
Bicycle rider	4 (2%)	6 (8%)	28 (11%)	33 (10%)	5 (3%)	
Pedestrian	3 (2%)	3 (4%)	30 (12%)	23 (7%)	13 (9%)	
Missing	0 (0%)	0 (0%)	8 (3%)	7 (2%)	1 (1%)	
Hospital admission	51 (31%)	34 (44%)	131 (52%)	159 (46%)	57 (38%)	0.10
Risk stratification						
Low (OMPSQ ≤50)	83 (50%)	32 (41%)	145 (57%)	195 (56%)	65 (43%)	< 0.01
High (OMPSQ > 50)	37 (22%)	35 (45%)	55 (22%)	73 (21%)	54 (36%)	
Missing	46 (28%)	11 (14%)	54 (21%)	79 (23%)	32 (21%)	

Note: *OMPSQ* score from the Short Form - Orebro Musculoskeletal Pain Screening Questionnaire

### Health related quality of life at baseline

**Fig. 2:**
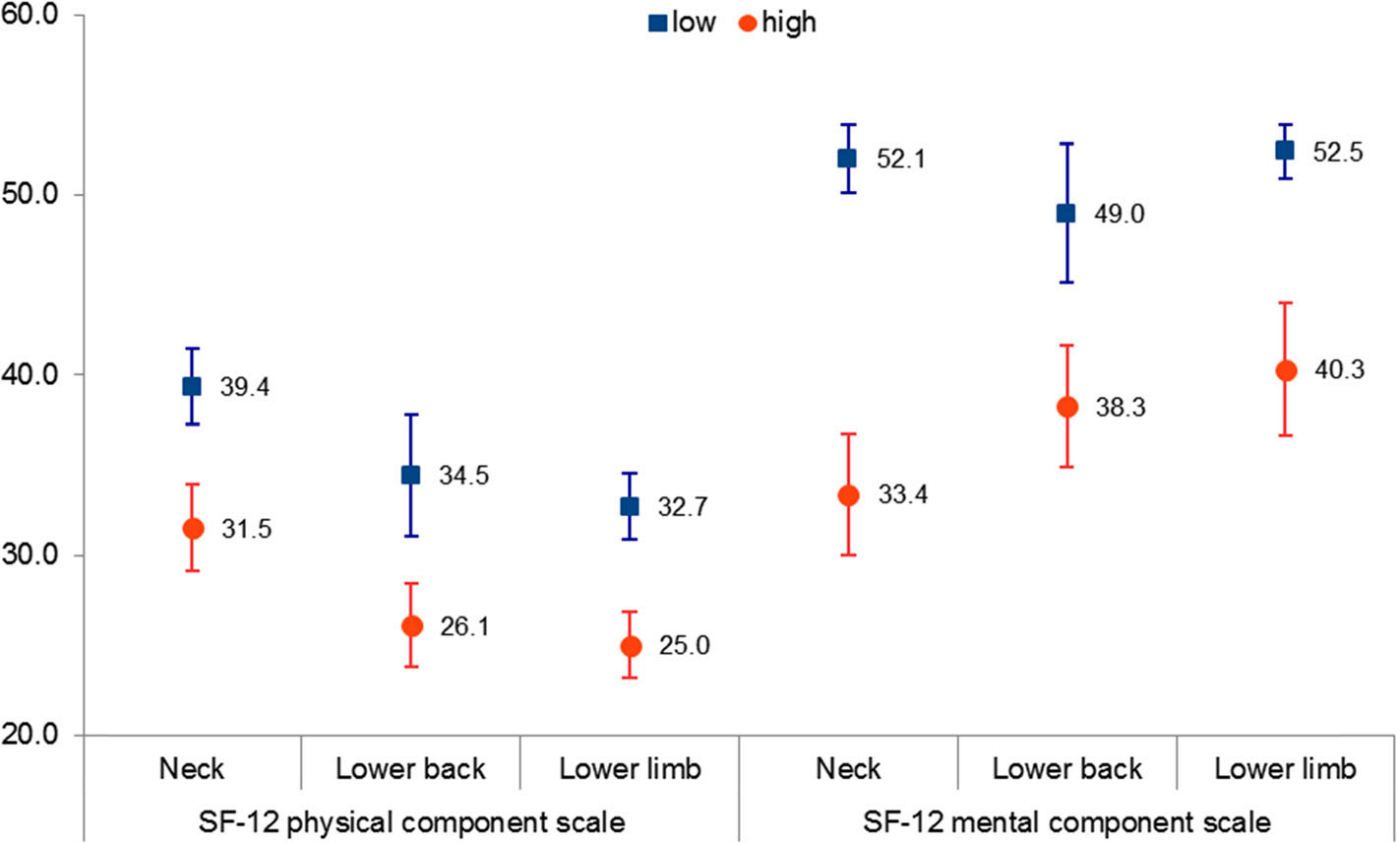
Mean and 95% CI of the SF-12 physical and mental component summary scale at baseline for neck, lower back and lower limb injuries stratified by low and high-risk using OMPSQ

At baseline, the within-group SF-12 physical and mental component summary scores for participants stratified as low-risk of non-recovery were significantly higher than those stratified as high-risk (*p* <  0.001, Fig. 2) . In terms of between-group comparisons, participants with neck injuries, both low and high-risk groups, had higher physical component scores than those with other injuries. Conversely, those with neck injuries stratified as high-risk of non-recovery had lower mental component scores than those with other injuries. However, none of these between-group differences were statistically significant.

### Outcomes at 6-month follow-up

Across all injury groups, significantly higher proportions of participants stratified as low-risk of non-recovery at baseline recovered (GPE ≥ 4) compared with those stratified as high-risk (Table 3). The adjusted likelihood of being recovered in the low-risk group was 2.45 to 3.08 times higher than those in the high-risk groups. Adjustments were made for participants’ age, self-rated general health prior to the injury, history of chronic illness prior to the injury and hospital admission status following the injury.

**Table 3:** Recovery at 6 months and adjusted risk ratio for recovery (GPE ≥ 4) by injury groups

Injury group	% Recovered		Unadjusted RR (95% CI)	Adjusted^a^ RR (95% CI)
	Low	High		
Neck (*n* = 77)	68.5%	26.1%	2.63 (1.29 to 5.35)	2.45 (1.18 to 5.12)
Lower back (*n* = 44)	56.5%	14.3%	3.96 (1.31 to 11.97)	3.03 (1.06 to 8.64)
Lower limb (*n* = 147)	61.9%	17.2%	3.59 (1.60 to 8.06)	3.08 (1.34 to 7.08)
**All (*n*** **= 268)**	63.1%	19.2%	3.29 (2.03 to 5.34)	2.96 (1.81 to 4.82)

Note: ^a^Adjusted risk ratio (RR), adjusting for participants’ age, self-rated general health prior to the injury, history of chronic illness prior to the injury and hospital admission status following the injury; *CI* Confidence interval

Similarly, significantly greater proportions of participants stratified as low-risk at baseline returned fully to pre-injury work compared with those stratified as high-risk (Table 4). Of these, the adjusted risk ratios of returning to work fully were statistically significant in neck (ARR = 1.68, 95% CI: 1.09 to 2.59) and lower back (ARR = 2.79, 95% CI: 1.17 to 6.68) injury groups.

**Table 4:** Returning fully to pre-injury work at 6 months and adjusted risk ratio of returning to work by injury groups

Injury group	% returning fully to pre-injury work		Unadjusted RR (95% CI)	Adjusted^a^ RR (95% CI)
	Low	High		
Neck (*n* = 55)	90.0%	60.0%	1.50 (0.98 to 2.31)	1.68 (1.09 to 2.59)
Lower back (*n* = 27)	84.6%	35.7%	2.37 (1.11 to 5.04)	2.79 (1.17 to 6.68)
Lower limb (*n* = 106)	92.3%	66.7%	1.38 (0.96 to 1.99)	1.35 (0.95 to 1.93)
**All (*n*** **= 188)**	91.0%	54.6%	1.67 (1.27 to 2.20)	1.65 (1.25 to 2.17)

^a^Adjusted risk ratio (RR), adjusting for participants’ age, self-rated general health prior to the injury, history of chronic illness prior to the injury and hospital admission status following the injury; *CI* Confidence interval

**Fig. 3:**
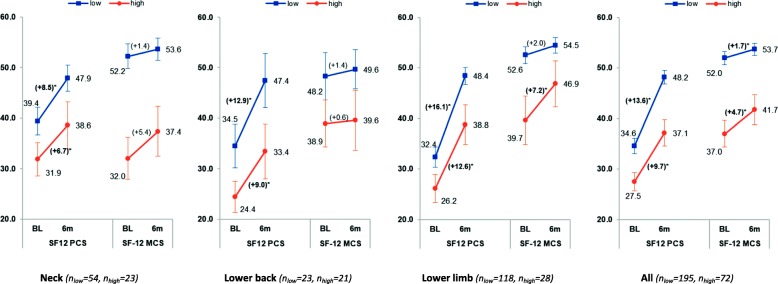
Mean and 95% CI for SF-12 physical and mental component summary scales at baseline and 6 month follow-up by injury groups. * Statistically significant in paired t-test comparing SF-12 scores (physical and mental) at baseline and 6 month follow-up

There were statistically significant improvements between baseline and 6-month follow-up scores for the SF12 physical component summary scale within all three injury groups and within low and high-risk groups (Fig. 3 and Table 5). The improvements were largest among those who sustained lower limb injury, followed by those who sustained lower back and neck injuries. Across all injury groups and all injuries combined, low-risk participants had larger improvements in the physical component scores (between baseline and 6-month follow-up) than high-risk participants. However, these differences were not statistically significant.

**Table 5:** Mean and change (95% CI) of SF-12 physical and mental component summary scales between baseline and 6-month follow-up

		Low risk			High risk		
		Baseline	6 months	Change (95% CI)	Baseline	6 months	Change (95% CI)
Physical score	Neck	39.4	47.9	8.5 (5.3 to 11.6)^a^	31.9	38.6	6.7 (1.9 to 11.5)^a^
	Lower back	34.5	47.4	12.9 (7.1 to 18.8)^a^	24.4	33.4	9.0 (2.8 to 15.1)^a^
	Lower limb	32.4	48.4	16.1 (14 to 18.1)^a^	26.2	38.8	12.6 (8.4 to 16.8)^a^
	All	34.6	48.2	13.6 (11.9 to 15.3)^a^	27.5	37.1	9.7 (6.9 to 14.2)^a^
Mental score	Neck	52.2	53.6	1.4 (−0.8 to 3.5)	32.0	37.4	5.4 (−0.2 to 10.9)
	Lower back	48.2	49.6	1.4 (−3.6 to 6.4)	38.9	39.6	0.6 (−6.0 to 7.3)
	Lower limb	52.6	54.5	2.0 (−0.1 to 4.0)	39.7	46.9	7.2 (2.6 to 11.9)^a^
	All	52.0	53.7	1.7 (0.3 to 3.2)^a^	37.0	41.7	4.7 (1.6 to 7.8)^a^

Note: ^a^Statistically significant; CI = Confidence interval

In terms of the SF12-mental component summary scale, while there seems to be improvements within injury groups as well as within risk levels, statistically significant improvements were only observed in the lower limb injury stratified as high-risk (+ 7.2, 95% CI: 2.6 to 11.9) and all injuries combined (low-risk: + 1.7, 95% CI: 0.3 to 3.2; and high-risk: + 4.7, 95% CI: 1.6 to 7.8). Between risk groups, the improvements were less in low-risk participants than those in high-risk participants (across all injury groups and all injuries combined); however such differences were also not statistically significant.

## Discussion

Our study investigated differences in recovery, return to work and health related quality of life at 6 months following a RTI between low and high-risk of non-recovery groups stratified by the SF-OMPSQ. We found statistically significant higher proportion of recovery and returning to work in the low-risk than the high-risk group. In the occupational injury setting, the Work Injury Screening and Early intervention (WISE) study showed positive outcomes from the application of the SF-OMPSQ in identifying and directing appropriate care and treatment for injured workers based on their identified risk level [9]. Our results, from an inception cohort study, indicated that the SF-OMPSQ is a promising generic tool to identify people at risk of poor recovery among those with musculoskeletal injury to the neck (whiplash), lower back or lower limb after a RTI. The SF-OMPSQ would not only work in the occupational injury setting but also in the cohort traumatic RTIs and across a number of common musculoskeletal injuries.

The significant differences in the SF-12 physical and mental summary component scales at baseline between risk group show that the SF-OMPSQ would also well discriminate people with poorer quality of life when they were identified as being at high-risk of non-recovery. This discriminative ability of the tool would be used by clinician to direct appropriate type and amount of care by the level of risk identified. Over time, the greater extent of improvements in physical scores (between baseline and 6 months after the injury) in the low-risk than those in the high-risk group across all injury groups suggest that regardless of body part injured, people with musculoskeletal pain after an RTI could be managed similarly using a stratified care model; more care would be directed to those identified at higher risk of non-recovery.

The strength in our study is that it demonstrated some promising properties of the SF-OMPSQ for RTIs. However, room for improvement of the tool still exists. In the low-risk of non-recovery group in our study, there were still large proportions of participants, who did not recover and similarly some of those with high-risk, who did recover. This suggests that a group with a medium risk of non-recovery may exist. In fact, a number of risk stratification tools for musculoskeletal injuries have medium level of non-recovery in addition to the common low and high-risk levels, such as the clinical prediction rule for whiplash injuries [14] or the Keele STarT Back Screening Tool for low back pain [7]. The identification of medium risk group would minimise the likelihood of missing out people who should have a more comprehensive care if they were stratified into the low-risk group.

Our study also has limitations. Participants’ recovery, return to work and/or health related quality of life would be influenced by other factors such as the medical care and treatment that they received following their injuries. At this stage, we are limited in the data collected from the study questionnaires. In the design of the original study, linked data on health service utilisation and hospitalisation for all participants will be available [22]. These additional data would allow us to conduct further analyses adjusting for health and hospital service utilisation. While we conducted multi-variable analyses when comparing the recovery and return to work outcomes to adjust for potential confounders, we were limited in variables/participants’ characteristics included in our questionnaires. Therefore, there was potential for residual confounding due to unmeasured factors such as personal coping skills, family circumstances, employer characteristics, working conditions, readiness/intent to return to work scales. The observed results for health-related quality of life were also potentially confounded due to measured factors, since they were not adjusted for in the analysis. Another limitation was the bias due to loss to follow-up and missingness in the data, which would influence our observed associations.

Despite limitations, results from our study provided some important implications for practice and further research. The tool was administered by trained interviewers, who were research nurses at study recruitment sites, within 28 days of the crash. In this and the WISE study, the tool was administered in a clinical or primary setting. It was demonstrated that the tool was better in these setting compared to the data from participants from the general public. The tool would potentially be tested by other users, such as an insurer case manager, for reliability and validity compared to clinicians. Another aspect is the time frame for the tool to be administered. A window of time between four and 6 weeks from the time of crash would be considered appropriate. Once the risk of poor recovery is identified, there would be time for intervention before the condition/pain would become chronic (i.e. lasting for 3 months or more). For patients identified as high-risk, they would be referred to specialist for further examination and appropriate care.

For future research, in addition to the study to identify threshold for medium risk group based on the SF-OMPSQ score, there should be studies to compare use of the SF-OMPSQ and other risk stratification tools. For instance, the SF-OMPSQ would be compared with the clinical prediction rule to stratify risk of non-recovery among whiplash injuries or the SF-OMPSQ would be compared with the Keele STarT Back Screening Tool to identify risk levels for patients with low back pain. In addition, a randomised trial similar to the WISE study for RTIs with care and treatment directed by the use of the SF-OMPSQ score would also be desirable.

## Conclusion

Our study provides evidence that the SF-OMPSQ could be used as a prognostic tool for early identification of people with risk of non-recovery following RTI. Consistently across common musculoskeletal injuries (including neck, lower back and lower limb), individuals identified to be at low-risk were more likely to recover and return to work. Further research is needed to compare the SF-OPMSQ and other prognostic tools for its reliability and validity; and also to examine its feasibility to apply in the hospital and primary health care settings, and then to drive appropriate level of care according to the level of risk identified.

## Data Availability

The datasets used and/or analysed in the current study are available upon reasonable request to Professor Ian D Cameron, Principal Investigator of the FISH study and Head of John Walsh Centre for Rehabilitation Research, the University of Sydney.

## Abbreviations

CI: Confidence interval
FISH study: Study on factors influencing social and health outcomes following road traffic injuries in New South Wales (NSW)
GPE: Global Perceived Effect
MCS: Mental Component Summary
NSW: New South Wales
PCS: Physical Component Summary
RR: Risk ratio
RTI: Road traffic injury
RTW: Return to work
SF-12: Short-form 12-item
SF-OMPSQ: Short form - Örebro Musculoskeletal Pain Screening Questionnaire
